# *LGR4* Gene Polymorphisms Are Associated With Bone and Obesity Phenotypes in Chinese Female Nuclear Families

**DOI:** 10.3389/fendo.2021.656077

**Published:** 2021-10-11

**Authors:** Su-qin Shi, Shan-shan Li, Xiao-ya Zhang, Zhe Wei, Wen-zhen Fu, Jin-wei He, Yun-qiu Hu, Miao Li, Li-li Zheng, Zhen-lin Zhang

**Affiliations:** ^1^ Department of Endocrinology, The First Affiliated Hospital of Henan University of Chinese Medicine, Zhengzhou, China; ^2^ Metabolic Bone Disease and Genetic Research Unit, Department of Osteoporosis and Bone Disease, Shanghai Jiao Tong University Affiliated Sixth People’s Hospital, Shanghai, China; ^3^ Department of Endocrinology, The First Affiliated Hospital of Zhengzhou University, Zhengzhou, China

**Keywords:** *LGR4*, SNP, osteoporosis, obesity, peak bone mineral density, fat mass, QTDT

## Abstract

**Objective:**

The current study was conducted to determine whether peak bone mineral density (BMD) and obesity phenotypes are associated with certain *LGR4* gene polymorphisms found in Chinese nuclear families with female children.

**Methods:**

A total of 22 single nucleotide polymorphisms (SNPs) located in and around the *LGR4* gene were identified in 1,300 subjects who were members of 390 Chinese nuclear families with female children. Then, BMD readings of the femoral neck, total hip, and lumbar spine as well as measurements of the total lean mass (TLM), total fat mass (TFM), and trunk fat mass were obtained *via* dual-energy X-ray absorptiometry. The quantitative transmission disequilibrium test was used to analyze the associations between specific SNPs and *LGR4* haplotypes and peak BMD as well as between *LGR4* haplotypes and TLM, percent lean mass, TFM, percent fat mass, trunk fat mass, and body mass index (BMI).

**Results:**

Here, rs7936621 was significantly associated with the BMD values for the total hip and lumbar spine, while rs10835171 and rs6484295 were associated with the trunk fat mass and BMI, respectively. Regarding the haplotypes, we found significant associations between GAA in block 2 and trunk fat mass and BMI, between AGCGT in block 3 and total hip BMD, between TGCTCC in block 5 and femoral neck BMD, and between TACTTC in block 5 and both lumbar spine and femoral neck BMD (all *P*-values < 0.05).

**Conclusion:**

Genetic variations of the *LGR4* gene are related to peak BMD, BMI, and trunk fat mass.

## Introduction

Osteoporosis and obesity are common occurrences during the aging process, and both are subject to environmental and genetic influences. Individuals with osteoporosis have a low bone mineral density (BMD) and an increased risk of fracture due to degradation of their bone microstructure. It is currently estimated that >200 million people have osteoporosis worldwide. In the United States, osteoporosis is an underlying cause of 1.3 million fractures, which cost $10 billion per year to repair (1). As a complex quantitative trait that is 50%–85% heritable, BMD can serve as a surrogate marker for osteoporosis (2–4). Obesity occurs due to an excessive accumulation of body fat and is closely associated with metabolic, cardiovascular, and physical disabilities (5). While body mass index (BMI) is the most commonly used index of obesity, it does not directly measure the total amount of body fat. Alternative ways of measuring obesity include measurements of the total fat mass, trunk fat mass, and percent body fat. Studies of twins and families in several populations have shown that BMI is strongly influenced by genetics and is 40%–70% inheritable (6).

While osteoporosis and obesity were once viewed as non-related disorders, they are now thought to be closely associated with each other. Increased body weight, as a form of mechanical loading, can induce osteogenesis and protect against bone loss (7). Mesenchymal stem cells (MSCs) in bone marrow give rise to both osteoblasts and adipocytes. Obesity induces the MSCs to generate greater numbers of adipocytes than osteoblasts, which leads to decreased bone formation (8). As an endocrine organ, adipose tissue secretes estrogen-synthesizing enzymes that increase estrogen levels. The resulting increased levels of estrogen then enhance osteoclast apoptosis (9) and inhibit osteoblast apoptosis (10), thereby decreasing bone tissue resorption. Moreover, adipose tissue also produces various cytokines and adipokines, whose dysregulation promotes a cascade of metabolic alterations that lead to bone loss (11, 12), possibly by creating and maintaining a low-grade inflammatory environment (13). Moreover, certain molecules produced by bone (e.g., osteopontin and osteocalcin) are known to help regulate body weight and maintain appropriate glucose levels (14). When examined at the genetic level, BMD and BMI share a 10%–20% genetic variation (15). Several studies have identified pleiotropic genes [e.g., *VDR*, *ESR1* (16), and *FTO* (17)] that assist in regulating both body composition and the osteoporosis process.

The gene that encodes the LGR4 protein, a leucine-rich protein also known as GPR48, maps to chromosome 11p14.1, which comprises 18 exons and 19 introns. The LGR4 protein consists of 951 amino acids, including 17 N-terminal leucine-rich repeats. The protein also contains a seven-transmembrane region (18). It contributes to the physiological function of several organs through the role it plays in both Wnt and cyclic AMP/protein kinase A (PKA) signaling (19, 20). A recent large-scale study conducted in Iceland identified a strong association between the nonsense mutation c.376C>T [p.R126Xc.367C-T; arg126 to ter; R126Xarg126 to ter; R126Xarg126 to ter; R126Xarg126 to ter; R126Xarg126 to ter; R126Xarg126 to ter; R126Xarg126 to ter; R126X)] found within the *LGR4* gene and both lower BMD values and osteoporotic fracture rates (21). Moreover, *LGR4* has been found to help control body weight by regulating the conversion of white fat into brown fat (22). Furthermore, a Chinese study that compared obese subjects with control subjects found that a heterogenous variant of c.2248G>A within *LGR4* is associated with obesity (23). These results suggest that *LGR4* can influence the phenotype of both osteoporosis and obesity. To investigate the validity of this hypothesis, we used the quantitative transmission disequilibrium test (QTDT) to examine whether *LGR4* polymorphisms are associated with variations in peak BMD and obesity-related phenotypes in a large cohort of female nuclear family members residing in China.

## Materials and Methods

### Subjects

The study protocol was reviewed and approved by the Shanghai Jiao Tong University Affiliated Sixth People’s Hospital Ethics Committee. Each study participant signed a written informed consent form prior to enrollment. The study participants resided in Shanghai City and were of Chinese Han ethnic descent.

From 2008 to 2015, we recruited 1,300 individuals who were members of 390 Chinese female-offspring nuclear families (24). The DNA samples obtained from 38 of the individuals were of poor quality and could not be sufficiently amplified to allow for discrimination of the genotypes. Thus, 1,262 individuals who were members of 379 different female nuclear families were included in our final analysis. Among them were 379 parents (mean age, 61.6 ± 7.5 years), 467 female children (mean age, 34.1 ± 6.8 years), and 37 male children (mean age, 34.6 ± 4.7 years). The families had a mean size of 3.33 individuals, as 268, 97, and 14 families included one, two, and three children, respectively. Each enrolled subject was asked to answer questions concerning their demographic characteristics, lifestyle, dietary habits, and tobacco/alcohol use. The study participant exclusion criteria were as follows: (1) significant health problems due to cerebral vascular disease; (2) diabetes; (3) chronic kidney, liver, or lung disease; (4) alcoholism; (5) pharmacologic doses of corticosteroids for >3 months; (6) anticonvulsant therapy for >6 months; (7) evidence of other hereditary or metabolic bone diseases; (8) rheumatoid arthritis or a collagen disease; (9) major gastrointestinal disease within the last year; (10) any major endocrinopathy that would affect bone mass such as hyperthyroidism, hypogonadism, or hypercortisolism; (11) any neurological or musculoskeletal condition that might cause secondary low bone mass (16, 24–30).

### Phenotype Measurements

A dual-energy X-ray absorptiometry (DXA) densitometer manufactured by Lunar Corporation (Madison, WI, U.S.A.) and operated in fan-beam mode was used to measure the bone density in the lumbar spine (L1–4) and left proximal femur (including the femoral neck and total hip) as well as to obtain measurements of the total lean mass (TLM), total fat mass (TFM), and trunk fat mass. The individual ratios of TFM to body weight and TLM to body weight were used as measurements of the percent fat mass (PFM) and percent lean mass (PLM), respectively. The densitometer was calibrated on a daily basis. Fifteen different individuals were each measured three times, and the results were used to calculate the coefficients of variability (CVs). The CV values of the measurements for the lumbar spine, femoral neck, total hip, and trochanter were 1.39%, 2.22%, 0.7%, and 1.41%, respectively. With regard to body composition, the CV values for the TLM, TFM, and trunk fat mass were 1.18%, 3.72%, and 2.52% respectively (28). The weekly phantom measurements taken during the course of our study showed that the DXA data had a reproducibility of 0.45% (16, 28). Standard methods were used to measure each subject’s height and weight, and the BMI was calculated as the weight in kilograms divided by the square of the height in meters.

### Single Nucleotide Polymorphism Selection and Genotyping

The 376 C>T mutation sites and 21 tagSNPs located in and around the *LGR4* gene were chosen for evaluation. The tagSNPs were selected based on information contained in the HapMap and dbSNP databases as well as factors that included: (1) whether the tag SNP had been validated in a Chinese population; (2) the amount of heterozygosity (minor allele frequency [MAF] >0.05); (3) whether the pairwise linkage disequilibrium (LD) of the algorithm bin tagSNP had an r^2^ value >0.8; (4) the reported functional importance of the tagSNP. Each study subject fasted overnight, after which a blood sample (5 mL) was taken and treated with disodium ethylenediaminetetraacetic acid. A chloroform–phenol solution was used to extract genomic DNA from peripheral blood leukocytes. Genotyping was performed with the combined use of an imLDR Multiplex Kit (Genesky Biotechnologies, Shanghai), an RT-PCR system (Mx300p; Stratagene, La Jolla, CA, U.S.A.), and the GeneMapper 4.1 software (Applied Biosystems, Foster City, CA, U.S.A.).

### Haplotype and LD Analysis

A process described by Stephens et al. (2001) was used in combination with the PHASE software (version 2.1) (31) to obtain haplotypes from the study population’s genotype data. The Haploview software (version 4.2) was used to assess the significance of the LD that existed between the *LGR4* gene markers (32). We also examined Lewontin’s D’ and LD coefficients (r^2^) between all pairs of biallelic loci. The genotype and haplotype frequencies were determined using genotype information obtained from non-related parents in the nuclear families.

### Statistical Analysis

The genotype occurrences of each of the 22 polymorphic sites identified in the parents in each nuclear family were analyzed with the χ^2^ test to assess their conformity to the Hardy–Weinberg equilibrium. The QTDT (orthogonal model) was performed to evaluate the parameters of population stratification, total association, linkage, and within-family associations between the various SNPs, haplotypes, BMD phenotypes, and obesity-related phenotypes. This method of using the QTDT to analyze data relevant to quantitative traits in conjunction with genotype data obtained from siblings and parents is very effective and was implemented herein using QTDT software (33). All children in the nuclear families were daughters, and the possible effects of the parental phenotypes were not analyzed by the QTDT; therefore, we did not use sex as a covariate when adjusting for variations in phenotype. Instead, the raw BMD values were adjusted using covariates that included height, age, and weight, and age was used to adjust the obesity phenotypes. To avoid false-positive results being obtained after multiple tests, the QTDT software package was used to perform 1,000 Monte Carlo permutations to obtain *P-*values that could be used to determine the accuracy of our results (34–36). The statistical power for each SNP is 0.8. A *P*-value <0.05 was shown to be statistically significant for all our analyses. The QTDT software package used can be downloaded at http://www.sph.umich.edu/csg/abecasis/QTDT/.

## Results

### Clinical Characteristics

This study enrolled 1,262 subjects who were members of 379 nuclear families with female children. The subjects included 758 parents and 467 female offspring. As we did not use the QTDT to analyze the effects of parental phenotypes, only the body compositions of the daughters were obtained. The characteristics of the enrolled subjects are listed in **Table 1**.

**Table 1:** The basic characteristics of the female-offspring nuclear families (mean ± SD).

Variables	Father	Mather	Daughter
	(n = 379)	(n = 379)	(n = 467)
Age (years)	62.9 ± 7.8	60.4 ± 7.0	34.1 ± 6.8
Height (cm)	167.0 ± 6.0	155.7 ± 5.6	160.1 ± 5.2
Weight (Kg)	69.9 ± 9.8	59.5 ± 8.9	56.1 ± 8.6
Lumbar spine BMD (g/cm^2^)	1.136 ± 0.185	0.995 ± 0.165	1.177 ± 0.136
Femoral neck BMD (g/cm^2^)	0.889 ± 0.122	0.797 ± 0.124	0.935 ± 0.122
Total hip BMD (g/cm^2^)	0.959 ± 0.127	0.856 ± 0.130	0.966 ± 0.120
BMI (Kg/m^2^)	25.0 ± 3.1	24.5 ± 3.5	21.9 ± 3.2
Total fat mass (Kg)	–	–	17.46 ± 6.07
Total lean mass (Kg)	–	–	35.94 ± 3.73
PFM (%)	–	–	0.30 ± 0.06
PLM (%)	–	–	0.65 ± 0.06
trunk fat mass (Kg)	–	–	9.16 ± 3.54

BMD, bone mineral density; BMI, body mass index; PFM, percentage of fat mass; PLM, percentage of lean mass.

### SNP Characterization and LD

We initially examined 22 SNPs located within and around *LGR4*. Because our genotype analysis showed that only one C376T genotype (GG) was present, C376T was excluded from our final statistical analysis. Moreover, rs11029986 had a MAF <0.05 and was also excluded. However, 20 SNPs had a MAF ≥0.07 and displayed Hardy–Weinberg equilibrium. Data obtained from 758 unrelated nuclear family parents were used to calculate the MAF values of the SNPs examined in our study. Detailed information concerning the SNPs is provided in **Table 2**.

**Table 2:** Information of the analyzed *LGR4* SNPs in this study.

SNP	Physical position	Locationand function	Allele change	Amino acid change	HWE*p*	MAF in dbSNP	MAF in this study
rs2447995	27388910	3’-UTR	T>C	NA	0.53	0.15	0.14
rs1531557	27392574	Intron	C>T	NA	0.73	0.13	0.11
rs10835171	27398845	Intron	A>G	NA	0.20	0.44	0.45
rs10835173	27401190	Intron	A>G	NA	0.68	0.40	0.48
rs11029986	27401389	Intron	C>T	NA	1.0	0.12	0.04
rs6484295	27406844	synon codon	G>A	S191S	0.14	0.43	0.42
rs2219783	27411298	Intron	A>G	NA	0.71	0.09	0.07
C376T	27412666	nonsense mutant	G/G	/	1.0	/	0.0
rs7936621	27426391	Intron	G>A	NA	1.0	0.29	0.30
rs12787344	27428264	Intron	C>A	NA	0.08	0.13	0.19
rs7927234	27436356	Intron	G>C	NA	0.87	0.04	0.08
rs4128868	27444517	Intron	T>C	NA	0.11	0.21	0.26
rs4514364	27456059	Intron	T>C	NA	0.21	0.22	0.25
rs4074516	27465591	Intron	T>C	NA	0.30	0.48	0.45
rs4923445	27471596	Intron	A>G	NA	0.81	0.20	0.22
rs4542364	27473981	Intron	A>G	NA	1.0	0.32	0.40
rs11030014	27480827	Intron	T>C	NA	0.13	0.09	0.18
rs16917037	27483834	Intron	G>A	NA	0.96	0.22	0.21
rs11030016	27487992	Intron	T>C	NA	1.0	0.44	0.46
rs12796247	27494625	Intron	C>T	NA	0.74	0.37	0.37
rs4923447	27495259	Intron	T>C	NA	0.53	0.13	0.16
rs10835187	27505677	/	T>C	NA	0.95	0.46	0.42

NA, not applicable; SNP, single-nucleotide polymorphism; HWE, Hardy-Weinberg equilibrium; MAF, minor allele frequency.

**Figure 1:**
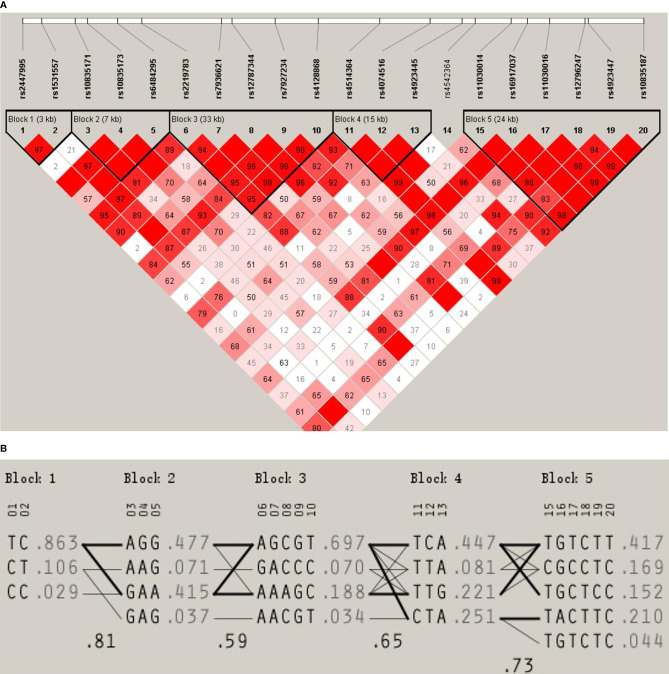
**(A)** LD pattern for the *LGR4* gene. The LD block structure is described by Haploview. From white to red, the increase in color indicates an increase in the strength of the LD. The values in the cells are the pairwise degrees of the LD. **(B)** Haplotype frequencies of the *LGR4* gene.

We used the D’ values obtained from Haploview to identify five LD blocks in our study population, which ranged from 3 to 33 kb in size. The SNP rs4542364 had little LD with any of the other SNPs and, thus, could not be assigned to any block. The LD patterns of blocks 1–5 are shown in **Figure 1**. The blocks had D’ values ranging from 0.93 to 1.0. The results of our analysis using the PHASE software suggested the presence of different haplotypes in our population. The frequencies of haplotypes >1% were counted. The frequencies of haplotypes TC, CT, and CC in block 1 were 87.0%, 10.3%, and 2.5%, respectively (*D*′ = 0.978, *r* ^2^ = 0.744), those of haplotypes AGG, GAA, AAG, and GAG in block 2 were 47.4%, 42.0%, 6.9%, and 3.6%, respectively (*D*′ = 1, 0.647 ≤ *r* ^2^ ≤ 0.861), and those of haplotypes AGCGT, AAAGC, GACCC, and AACGT in block 3 were 69.6%, 18.8%, 6.8%, and 3.4%, respectively (0.949 ≤ *D*′ ≤ 1, 0.018 ≤ *r* ^2^ ≤ 0.86). Moreover, those of haplotypes TCA, CTA, TTG, and TTA in block 4 were 44.4%, 25.6%, 21.7%, and 8.3%, respectively (*D*′ = 1, 0.095 ≤ *r* ^2^ ≤ 0.27), and those of haplotypes TGTCTT, TACTTC, CGCCTC, TGCTCC, and TGTCTC in block 5 were 41.4%, 21.3%, 16.7%, 15.0%, and 4.6%, respectively (0.837 ≤ *D*′ ≤ 1, 0.028 ≤ *r* ^2^ ≤ 0.828).

### Association Between SNPs and Peak BMD/Body Composition

We used the QTDT program to analyze the association between the SNPs present in *LGR4* and the peak BMD and obesity phenotypes in our female-offspring nuclear families. Totals of 152, 126, 281, 281, 285, 92, 238, 200, 96, 238, 215, 263, 215, 268, 178, 205, 274, 263, 171, and 272 informative nuclear families were included in our QTDT analyses of rs2447995, rs1531557, rs10835171, rs10835173, rs6484295, rs2219783, rs7936621, rs12787344, rs7927234, rs4128868, rs4514364, rs4074516, rs4923445, rs4542364, rs11030014, rs16917037, rs11030016, rs12796247, rs4923447, and rs1083518, respectively. At least one parent who was heterozygous was present in each of those families. The significant *P-*values of the SNPs obtained from the QTDT are shown in **Table 3**, and the non-significant *P-*values of the SNPs are shown in **Table S1**. We found evidence of population stratification for rs4074516 and femoral neck BMD (*P =* 0.048), rs4542364 and trunk fat mass (*P =* 9.00E-29), rs11030014 and lumbar spine BMD (*P =* 0.04), rs4923447 and TFM, PFM, and PLM (*P =* 0.041, 0.013, and 0.017, respectively), and rs10835187 and PFM and PLM (*P =* 0.040 and 0.03, respectively). Total associations were found between rs2447995 and trunk fat mass (*P =* 7.00E-29), rs10835173 and trunk fat mass (*P =* 7.00E-29), rs7936621 and femoral neck and total hip BMD and trunk fat mass (*P =* 0.003, 0.013, and 9.00E-29, respectively), rs1278734 and femoral neck BMD and trunk fat mass (*P =* 0.025 and 9.00E-29, respectively), rs1103001 and femoral neck, total hip, and lumbar spine BMD and trunk fat mass (*P =* 0.011, 0.024, 0.037, and 9.00E-29, respectively), rs16917037 and femoral neck BMD (*P =* 0.037), and rs10835187 and femoral neck and total hip BMD (*P =* 0.032 and 0.015, respectively). Our analyses conducted to detect within-family associations found significant associations between rs2447995 and trunk fat mass (*P =* 6.00E-29), rs10835173 and trunk fat mass (*P =* 6.00E-29), rs7936621 and femoral neck, total hip, and lumbar spine BMD (*P =* 0.031, 0.020, and 0.017, respectively), rs4128868 and trunk fat mass (*P =* 7.00E-29), and rs11030014 and trunk fat mass (*P =* 1.00E-28). To avoid bias caused by multiple tests, the *P*-values calculated for the within-family associations were obtained after performing 1,000 Monte Carlo permutations. After those permutations were completed, rs7936621 remained significantly associated with the BMD values for the femoral neck, total hip, and lumbar spine (*P =* 0.038, 0.029, and 0.031, respectively), while rs10835171 and rs6484295 were significantly associated with trunk fat mass (*P =* 0.045) and BMI (*P =* 0.043).

**Table 3:** QTDT results regarding the associations between the single nucleotide polymorphisms and phenotypic variations.

Genotype	rs2447995	rs10835171	rs10835173	rs6484295	rs7936621	rs12787344	rs4128868	rs4074516	rs4542364	rs11030014	rs16917037	rs4923447	rs10835187
Test of population stratification													
Femoral neck BMD	0.707	0.892	0.758	0.880	0.972	0.661	0.398	**0.048**	0.317	0.062	0.896	0.247	0.477
Total hip BMD	0.649	0.839	0.832	0.949	0.445	0.522	0.877	0.139	0.721	0.148	0.947	0.292	0.657
Lumbar spine BMD	0.295	0.668	0.359	0.869	0.141	0.693	0.470	0.112	0.313	**0.040**	0.207	0.048	0.231
TFM	0.635	0.233	0.718	0.564	0.945	0.674	0.731	0.740	1.000	0.553	0.691	**0.041**	0.073
TLM	0.801	0.508	0.598	0.821	0.662	0.725	0.641	0.315	0.676	0.491	0.525	0.098	0.563
PFM	0.661	0.496	0.990	0.863	0.686	0.511	0.896	0.652	0.973	0.546	0.962	**0.013**	**0.040**
PLM	0.632	0.258	0.632	0.503	0.992	0.784	0.822	0.623	0.713	0.647	0.735	**0.017**	**0.030**
Trunk fat mass	0.557	0.164	0.528	0.412	0.600	1.000	0.448	0.774	**9.00E-29**	0.621	0.387	0.088	0.050
BMI	0.927	0.118	0.265	0.264	0.668	0.888	0.513	0.912	0.417	0.512	0.233	0.703	0.194
Tests for total association													
Femoral neck BMD	0.189	0.684	0.669	0.722	**0.003**	**0.025**	**0.025**	0.927	0.750	**0.011**	**0.037**	0.165	0.032
Total hip BMD	0.381	0.994	0.897	0.525	**0.013**	0.068	0.066	0.816	0.775	**0.024**	0.195	0.113	0.015
Lumbar spine BMD BMD	0.969	0.836	0.899	0.718	0.058	0.109	0.208	0.570	0.269	**0.037**	0.169	0.470	0.084
TFM	0.569	0.316	0.487	0.224	0.867	0.576	0.813	0.121	0.637	0.907	0.855	0.883	0.790
TLM	0.193	0.350	0.403	0.157	0.272	0.333	0.199	0.497	0.647	0.146	0.630	0.641	0.591
PFM	0.766	0.494	0.806	0.469	0.633	0.235	0.748	0.196	0.552	0.783	0.941	0.715	0.974
PLM	0.827	0.372	0.647	0.400	0.944	0.542	0.892	0.283	0.762	0.856	0.983	0.898	0.919
Trunk fat mass	**7.00E-29**	0.208	**7.00E-29**	0.135	**9.00E-29**	0.747	**9.00E-29**	0.223	1.000	**9.00E-29**	0.7162	0.805	0.623
BMI	0.389	0.230	0.377	0.131	0.431	0.778	0.382	0.106	0.704	0.813	0.738	0.673	0.993
Test for within-family association													
Femoral neck BMD	0.521	0.829	0.914	0.713	**0.031**	0.157	0.258	0.166	0.647	0.618	0.096	0.071	0.311
Total hip BMD	0.772	0.899	0.964	0.658	**0.020**	0.071	0.137	0.245	0.968	0.563	0.346	0.061	0.159
Lumbar spine BMD BMD	0.434	0.662	0.600	0.867	**0.017**	0.140	0.155	0.518	0.915	0.982	0.062	0.059	0.691
TFM	0.458	0.124	0.454	0.193	0.892	0.485	0.681	0.177	0.720	0.740	0.704	0.130	0.263
TLM	0.481	0.255	0.331	0.222	0.276	0.337	0.205	0.847	0.968	0.591	0.436	0.136	0.430
PFM	0.601	0.336	0.863	0.508	0.533	0.183	0.743	0.209	0.683	0.536	0.930	0.047	0.141
PLM	0.618	0.157	0.509	0.283	0.954	0.522	0.802	0.262	0.973	0.653	0.815	0.080	0.144
Trunk fat mass	**6.00E-29**	0.063	**6.00E-29**	0.094	**8.00E-29**	0.767	**7.00E-29**	0.348	0.829	**1.00E-28**	0.395	0.199	0.270
BMI	0.515	0.052	0.159	0.061	0.392	0.762	0.278	0.271	0.767	0.760	0.296	0.570	0.356
P1000 permutation of within-family													
Femoral neck BMD	0.487	0.837	0.923	0.740	**0.038**	0.157	0.297	0.175	0.610	0.597	0.064	0.097	0.341
Total hip BMD	0.733	0.908	0.960	0.691	**0.029**	0.080	0.157	0.271	0.965	0.576	0.312	0.105	0.174
Lumbar spine BMD BMD	0.437	0.677	0.602	0.859	**0.031**	0.139	0.191	0.519	0.924	0.985	0.054	0.071	0.695
TFM	0.537	0.127	0.466	0.168	0.963	0.470	0.680	0.237	0.761	0.795	0.757	0.147	0.366
TLM	0.484	0.265	0.354	0.261	0.362	0.380	0.269	0.877	0.957	0.591	0.404	0.215	0.411
PFM	0.618	0.309	0.862	0.478	0.458	0.104	0.675	0.227	0.711	0.534	0.930	0.056	0.135
PLM	0.628	0.117	0.462	0.244	0.933	0.448	0.770	0.263	0.973	0.643	0.821	0.067	0.138
Trunk fat mass	0.279	**0.045**	0.233	0.064	0.452	0.802	0.382	0.328	0.877	0.845	0.392	0.155	0.292
BMI	0.537	0.050	0.165	**0.043**	0.394	0.739	0.267	0.325	0.792	0.797	0.317	0.600	0.401

Bold indicates a significant P-value (<0.05).

### Association Between Haplotypes and Peak BMD/Body Composition

The genotype data obtained from our SNP analysis were used to perform a haplotype analysis that identified groups of three, four, four, four, and five haplotypes in blocks 1, 2, 3, 4, and 5, respectively; these haplotypes accounted for 99.8%, 99.9%, 98.6%, 100%, and 99.0%, respectively, of the unrelated parents in the blocks. We next used the QTDT program to examine how different haplotypes might be associated with peak BMD and obesity phenotypes. Totals of 43, 126, and 149 informative families were used for the QTDT of haplotypes CC, CT, and TC in block 1, respectively; 48, 288, 284, and 90 informative families were used for the QTDT of haplotypes GAC, GAA, AGG, and AAG in block 2, respectively; and 240, 45, 204, and 86 informative families were used for the QTDT of haplotypes AGCGT, AACGT, AAAGC, and GACCC in block 3, respectively, Moreover, 215, 112, 215, and 267 informative families were used for the QTDT of haplotypes CTA, TTA, TTG, and TCA in block 4, respectively, and 163, 57, 277, 205, and 174 informative families were used for the QTDT of haplotypes TGCTCC, TGTCTC, TGTCTT, TACTTC, and CGCCTC in block 5, respectively. Haplotype TGTCTT in block 5 showed significant degrees of population stratification with TFM, PFM, PLM, and trunk fat mass (*P =* 0.022, 0.014, 0.011, and 0.023, respectively). We also found significant total associations between trunk fat mass and haplotypes CC, CT, and TC in block 1 (*P =* 1.00E-28, 9.00E-29, and 6.00E-29, respectively), between AGCGT in block 3 and both femoral neck and total hip BMD (*P =* 0.009 and 0.026, respectively), between TGTCTT and total hip BMD (*P =* 0.029), between TACTTC and femoral neck BMD (*P =* 0.027), and between CGCCTC and femoral neck, total hip, and lumbar spine BMD in block 5 (*P =* 0.013, 0.031, and 0.038, respectively). Significant within-family associations were detected between trunk fat mass and haplotypes CC, CT, and TC in block 1 (*P =* 1.00E-28, 7.00E-29, and 5.00E-29, respectively), between GAA in block 2 and both BMI and trunk fat mass (*P =* 0.031 and 0.043, respectively), between AGCGT in block 3 and total hip BMD (*P =* 0.032), between GACCC in block 3 and trunk fat mass (*P =* 0.049), between TTA in block 4 and TFM (*P =* 0.033), between TGCTCC in block 5 and both femoral neck and total hip BMD (*P =* 0.015 and 0.041, respectively), and between TACTTC in block 5 and lumbar spine BMD (*P =* 0.040). Furthermore, after 1,000 permutations had been performed, significant within-family associations remained between GAA in block 2 and both trunk fat mass and BMI (*P =* 0.027 and 0.019, respectively), between AGCGT in block 3 and total hip BMD (*P =* 0.043), between TGCTCC in block 5 and femoral neck BMD (*P =* 0.025), and between TACTTC in block 5 and both femoral neck and lumbar spine BMD (*P =* 0.024 and 0.037, respectively). In contrast, no significant within-family associations after 1,000 permutations were found in either block 1 or block 4. The significant haplotype *P-*values obtained with the QTDT are listed in **Table 4**, and the non-significant *P-*values are listed in **Table S2**.

**Table 4:** QTDT results for the associations between the haplotype and phenotype variations.

	block 1			block 2	block 3		block 4	block 5			
	CC	CT	TC	GAA	AGCGT	GACCC	TTA	TGCTCC	TGTCTT	TACTTC	CGCCTC
Test for population stratification											
Femoral neck BMD	0.546	0.816	0.558	0.863	0.902	0.397	0.813	0.088	0.285	0.696	0.085
Total hip BMD	0.593	0.777	0.472	0.890	0.442	0.490	0.913	0.202	0.389	0.765	0.163
Lumbar spine BMD	0.581	0.214	0.560	0.799	0.245	0.482	0.551	0.067	0.232	0.137	0.061
TFM	0.933	0.522	0.476	0.435	0.635	0.051	0.126	0.140	**0.022**	0.662	0.814
TLM	0.869	0.879	0.931	0.856	0.613	0.481	0.661	0.078	0.693	0.590	0.395
PFM	0.816	0.621	0.537	0.655	0.975	0.124	0.357	0.056	**0.014**	0.994	0.902
PLM	0.767	0.651	0.534	0.403	0.669	0.178	0.461	0.054	**0.011**	0.658	0.975
Trunk fat mass	0.919	0.504	0.428	0.322	0.409	0.053	0.420	0.208	**0.023**	0.371	0.861
BMI	0.864	0.737	0.782	0.204	0.474	0.206	0.513	0.938	0.107	0.249	0.653
Tests for total association											
Femoral neck BMD	0.938	0.187	0.223	0.678	**0.009**	0.675	0.115	0.084	0.053	**0.027**	**0.013**
Total hip BMD	0.671	0.289	0.391	0.476	**0.026**	0.695	0.317	0.108	**0.029**	0.139	**0.031**
Lumbar spine BMD	0.818	0.829	0.882	0.675	0.167	0.751	0.298	0.739	0.132	0.151	**0.038**
TFM	0.886	0.495	0.336	0.108	0.804	0.437	0.137	0.736	0.753	0.906	0.755
TLM	0.377	0.277	0.181	0.131	0.205	0.635	0.271	0.806	0.568	0.700	0.189
PFM	0.848	0.603	0.475	0.317	0.860	0.337	0.188	0.918	0.960	0.964	0.893
PLM	0.920	0.694	0.524	0.271	0.853	0.358	0.222	0.703	0.888	0.951	0.953
Trunk fat mass	**1.00E-28**	**9.00E-29**	**6.00E-29**	0.072	0.525	0.380	1.000	0.716	0.522	0.709	0.485
BMI	0.806	0.341	0.260	0.077	0.293	0.419	0.246	0.973	0.875	0.827	0.593
Test for within-family association											
Femoral neck BMD	0.694	0.439	0.695	0.670	0.072	0.776	0.314	**0.015**	0.521	0.052	0.591
Total hip BMD	0.496	0.570	0.953	0.653	**0.032**	0.853	0.415	**0.041**	0.335	0.186	0.591
Lumbar spine BMD	0.573	0.304	0.596	0.879	0.071	0.796	0.241	0.128	0.803	**0.040**	0.897
TFM	0.851	0.351	0.242	0.084	0.598	0.055	**0.033**	0.418	0.165	0.708	0.966
TLM	0.628	0.524	0.410	0.205	0.208	0.404	0.266	0.155	0.495	0.520	0.740
PFM	0.968	0.473	0.349	0.294	0.917	0.078	0.110	0.204	0.091	0.976	0.855
PLM	0.881	0.549	0.374	0.167	0.667	0.110	0.161	0.276	0.091	0.740	0.949
Trunk fat mass	**1.00E-28**	**7.00E-29**	**5.00E-29**	**0.043**	0.308	**0.049**	1.000	0.457	0.234	0.413	0.716
BMI	0.973	0.367	0.343	**0.031**	0.212	0.143	0.195	0.974	0.301	0.352	0.961
P1000 permutation of within-family											
Femoral neck BMD	0.650	0.424	0.669	0.692	0.081	0.771	0.281	**0.025**	0.539	**0.024**	0.582
Total hip BMD	0.435	0.546	0.950	0.654	**0.043**	0.832	0.372	0.094	0.330	0.151	0.626
Lumbar spine BMD	0.513	0.324	0.592	0.870	0.093	0.820	0.208	0.157	0.791	**0.037**	0.901
TFM	0.836	0.471	0.325	0.070	0.616	0.154	0.084	0.427	0.263	0.745	0.947
TLM	0.493	0.548	0.419	0.243	0.296	0.448	0.278	0.237	0.475	0.493	0.746
PFM	0.967	0.500	0.354	0.261	0.898	0.080	0.131	0.204	0.102	0.973	0.851
PLM	0.881	0.567	0.382	0.117	0.609	0.118	0.175	0.251	0.105	0.754	0.951
Trunk fat mass	0.644	0.305	0.164	**0.027**	0.297	0.090	0.919	0.439	0.272	0.415	0.759
BMI	0.966	0.423	0.365	**0.019**	0.209	0.211	0.252	0.981	0.363	0.385	0.982

Bold indicates a significant P-value (<0.05).

## Discussion

Few studies have examined the association between *LGR4* gene polymorphisms and osteoporosis and obesity phenotypes. In 2013, Styrkarsdottir et al. (21) reported the presence of a nonsense mutation [c.376C>T (p.R126X)] in the *LCR4* gene of an Icelandic population. This mutation resulted in the production of a truncated and non-functional LGR4 protein, with the resultant physiological effect being a reduction in peak bone mass instead of an increased rate of bone loss, related to increasing age. Luo et al. (37) found that *LGR4^-/-^* and *LGR4* CKO mice exhibit low levels of BMD, and a subsequent morphometric study showed that the mean number, size, and surface area of the mouse osteoclasts as well as the amounts of eroded bone surface area in those mice were all significantly higher compared with those parameters in the control mice. These investigators also proposed that LGR4 and RANK compete for RANKL binding sites on osteoclasts; thus, LGR4 inhibits osteoclast differentiation and bone tissue remodeling. In contrast, LGR4 appears to function differently in osteoblasts, where it acts through the cAMP-PKA-CREB pathway to regulate ATF4 expression levels. Thus, LGR4 promotes both the differentiation of osteoblasts and the formation of new bone tissue (38). Based on those studies, we focused on examining whether *LGR4* SNPs could affect BMD in humans. However, none of our study subjects carried the c.376C>T mutation, consistent with results reported by Zou et al. (23). Our study found significant associations between the polymorphism rs7936621 and the BMD values for the femoral neck, total hip, and lumbar spine. An examination of the haplotype associations in five blocks with BMD values revealed strong associations between haplotype AGCGT in block 3 and total hip BMD, haplotype TGCTCC in block 5 and femoral neck BMD, and haplotype TACTTC in block 5 and both femoral neck and lumbar spine BMD. Based on this biological and statistical evidence, we propose that *LGR4* plays a role in regulating BMD or the osteoporotic process.

Additionally, an *in vitro* study showed that *LGR4* ablation potentiates the white-to-brown fat transition that occurs in areas of visceral fat. Meanwhile, a significant correlation has been identified between the *LGR4* c.2248G>A variant and both the waist circumference and waist-to-height ratio of young individuals as well as the amount of abdominal visceral fat in young obese subjects (23). Our study identified an association between rs10835171 and trunk fat mass as well as between rs6484295 and BMI, and our Haploview analysis showed that both of these SNPs were located in block 2. We also found that haplotype GAA in block 2 displayed significant within-family associations with trunk fat mass and BMI. Moreover, rs10835172, which is near rs10831571 and was found in block 2, has been reported to significantly correlate with BMI in Chinese individuals (22). In fact, we also observed a relationship between *LGR4* SNPs and appendicular fat mass but did not find any association between them. Therefore, we can suppose that either rs10835171 and rs6484295 by themselves or the region around them in block 2 may influence fat metabolism, especially central obesity, which is a known risk factor for both metabolic syndrome and type 2 diabetes. Further studies are required to elucidate how these SNPs may alter protein function and contribute to human central obesity.

This study has several strengths worth mentioning. First, to ensure a comprehensive analysis of the entire *LGR4* gene, we chose to study all 21 of its tagSNPs instead of only a small subset of the SNPs. Second, we chose peak BMD as the osteoporosis phenotype for examination, as this parameter is believed to be genetically controlled. Moreover, a larger number of phenotypes related to obesity were examined in this study than in previous studies. In addition to BMI, our study also examined other parameters including TFM, TLM, PFM, PLM, and trunk fat mass. A recent study showed that trunk fat mass as measured by DAX can be a reliable indicator of total abdominal fat (39), which is closely associated with various metabolic and cardiovascular diseases (38, 40). Third, due to the large number of Chinese nuclear families included in our study, our QTDT analysis was able to identify 281, 285, 288, 240, 205, and 163 informative nuclear families suitable for transition disequilibrium analysis at rs1083517, rs6484295, and haplotype GAA in block 2, haplotype AGCGT in block 3, TGCTCC in block 5, and haplotype TACTTC in block 5, respectively. This large sample size enhances the validity of our results. However, the study also has limitations that should be mentioned: First, DAX cannot distinguish between trunk subcutaneous and visceral mass. Second, information concerning the effects of environmental factors such as dietary fat intake and physical exercise, as well as the reproductive history (number of pregnancies, number of children and duration of breastfeeding), years since menopause, physical activity, estrogen replacement therapy or calcium supplements is lacking.

In conclusion, we identified common polymorphisms and haplotypes of *LGR4* that are associated with peak BMD, trunk fat mass, and BMI in young Chinese females.

## Data Availability Statement

The original contributions presented in the study are publicly available. This data can be found here: https://www.ncbi.nlm.nih.gov/SNP/snp_viewTable.cgi?handle=LGR4-SNP.

## Ethics Statement

The study protocol was reviewed and approved by the Shanghai Jiao Tong University Affiliated Sixth People’s Hospital Ethics Committee. Written informed consent to participate in this study was provided by the participants’ legal guardian/next of kin.

## Author Contributions

S-qS, L-lZ and Z-lZ conceived the idea and conceptualized the study. S-sL,X-yZ, ZW, W-zF and J-wH collected the data. ML and Y-qH analyzed the data. S-qS, L-lZ, and Z-lZ drafted the manuscript. All authors contributed to the article and approved the submitted version.

## Funding

This study was supported by the National Natural Science Foundation of China (81370978) and the Science and Technology Commission of Shanghai Municipality (16411954500). The funding body had no role in the design of the study and collection, analysis, and interpretation of data and in writing the manuscript.

## Conflict of Interest

The authors declare that the research was conducted in the absence of any commercial or financial relationships that could be construed as a potential conflict of interest.

## Publisher’s Note

All claims expressed in this article are solely those of the authors and do not necessarily represent those of their affiliated organizations, or those of the publisher, the editors and the reviewers. Any product that may be evaluated in this article, or claim that may be made by its manufacturer, is not guaranteed or endorsed by the publisher.

## References

[1] Nayak NK Khedkar CC Khedkar GD Khedkar CD. Osteoporosis. In: Caballero B Paul F Toldra F, editors. Encyclopedia of Food and Health. Oxford, Academic Press (2016). p. 181–5. Available at: https://www.sciencedirect.com/science/article/pii/B9780123849472005079.

[2] Slemenda CW Turner CH Peacock M Christian JC Sorbel J Hui SL. The Genetics of Proximal Femur Geometry, Distribution of Bone Mass and Bone Mineral Density. Osteoporos Int (1996) 6:178–82. doi: 10.1007/BF01623944 8704359

[3] Arden NK Baker J Hogg C Baan K Spector TD. The Heritability of Bone Mineral Density, Ultrasound of the Calcaneus and Hip Axis Length: A Study of Postmenopausal Twins. J Bone Miner Res (1996) 11:530–4. doi: 10.1002/jbmr.5650110414 8992884

[4] Smith DM Nance WE Kang KW Christian JC Johnston CC. Genetic Factors in Determining Bone Mass. J Clin Invest (1973) 52:2800–8. doi: 10.1172/JCI107476 PMC302548 4795916

[5] Haslam DW James WP. Obesit. Lancet (2005) 366:1197–209. doi: 10.1016/S0140-6736(05)67483-1 16198769

[6] Maes HH Neale MC Eaves LJ. Genetic and Environmental Factors in Relative Body Weight and Human Adiposity. Behav Genet (1997) 27:325–51. doi: 10.1023/A:1025635913927 9519560

[7] Hannan MT Felson DT Anderson JJ. Bone Mineral Density in Elderly Men and Women: Results From the Framingham Osteoporosis Study. J Bone Miner Res (1992) 7:547–53. doi: 10.1002/jbmr.5650070511 1615761

[8] Cao JJ. Effects of Obesity on Bone Metabolism. J Orthop Surg Res (2011) 6:30. doi: 10.1186/1749-799X-6-30 21676245 PMC3141563

[9] Kameda T Mano H Yuasa T Mori Y Miyazawa K Shiokawa M. Estrogen Inhibits Bone Resorption by Directly Inducing Apoptosis of the Bone-Resorbing Osteoclasts. J Exp Med (1997) 186:489–95. doi: 10.1084/jem.186.4.489 PMC2199029 9254647

[10] Bradford PG Gerace KV Roland RL Chrzan BG. Estrogen Regulation of Apoptosis in Osteoblasts. Physiol Behav (2010) 99:181–5. doi: 10.1016/j.physbeh.2009.04.025 PMC2825744 19426747

[11] Magni P Dozio E Galliera E Ruscica M Corsi MM. Molecular Aspects of Adipokine-Bone Interactions. Curr Mol Med (2010) 10:522–32. doi: 10.2174/1566524011009060522 20642443

[12] Tilg H Moschen AR. Inflammatory Mechanisms in the Regulation of Insulin Resistance. Mol Med (2008) 14:222–31. doi: 10.2119/2007-00119.Tilg PMC2215762 18235842

[13] Hotamisligil GS. Inflammation and Metabolic Disorders. Nature (2006) 444:860–7. doi: 10.1038/nature05485 17167474

[14] Gómez-Ambrosi J Rodríguez A Catalán V Frühbeck G. The Bone-Adipose Axis in Obesity and Weight Loss. Obes Surg (2008) 18:1134–43. doi: 10.1007/s11695-008-9548-1 18563500

[15] Deng FY Lei SF Li MX Jiang C Dvornyk V Deng HW. Genetic Determination and Correlation of Body Mass Index and Bone Mineral Density at the Spine and Hip in Chinese Han Ethnicity. Osteoporos Int (2006) 17:119–24. doi: 10.1007/s00198-005-1930-4 16025191

[16] Gu JM Xiao WJ He JW Zhang H Hu WW Hu YQ. Association Between VDR and ESR1 Gene Polymorphisms With Bone and Obesity Phenotypes in Chinese Male Nuclear Families. Acta Pharmacol Sin (2009) 30:1634–42. doi: 10.1038/aps.2009.169 PMC4007503 19960008

[17] Guo Y Liu H Yang TL Li SM Li SK Tian Q. The Fat Mass and Obesity Associated Gene, FTO, Is Also Associated With Osteoporosis Phenotypes. PloS One (2011) 6:e27312. doi: 10.1371/journal.pone.0027312 22125610 PMC3220685

[18] Loh ED Broussard SR Kolakowski LF. Molecular Characterization of a Novel Glycoprotein Hormone G-Protein-Coupled Receptor. Biochem Biophys Res Commun (2001) 282:757–64. doi: 10.1006/bbrc.2001.4625 11401528

[19] Weng J Luo J Cheng X Jin C Zhou X Qu J. Deletion of G Protein-Coupled Receptor 48 Leads to Ocular Anterior Segment Dysgenesis (ASD) Through Down-Regulation of Pitx2. Proc Natl Acad Sci USA (2008) 105:6081–6. doi: 10.1073/pnas.0708257105 PMC2329706 18424556

[20] Zhu C Zheng XF Yang YH Li B Wang YR Jiang SD. LGR4 Acts as a Key Receptor for R-Spondin 2 to Promote Osteogenesis Through Wnt Signaling Pathway. Cell Signal (2016) 28:989–1000. doi: 10.1016/j.cellsig.2016.04.010 27140682

[21] Styrkarsdottir U Thorleifsson G Sulem P Gudbjartsson DF Sigurdsson A Jonasdottir A. Nonsense Mutation in the LGR4 Gene Is Associated With Several Human Diseases and Other Traits. Nature (2013) 497:517–20. doi: 10.1038/nature12124 23644456

[22] Wang J Liu R Wang F Hong J Li X Chen M. Ablation of LGR4 Promotes Energy Expenditure by Driving White-to-Brown Fat Switch. Nat Cell Biol (2013) 15:1455–63. doi: 10.1038/ncb2867 24212090

[23] Zou Y Ning T Shi J Chen M Ding L Huang Y. Association of a Gain-of-Function Variant in LGR4 With Central Obesity. Obes (Silver Spring) (2017) 25:252–60. doi: 10.1002/oby.21704 27925416

[24] Wang C Hu YM He JW Gu JM Zhang H Hu WW. Association Between Low Density Lipoprotein Receptor-Related Protein 2 Gene Polymorphisms and Bone Mineral Density Variation in Chinese Population. PloS One (2011) 6:e28874. doi: 10.1371/journal.pone.0028874 22174918 PMC3235174

[25] Ke YH Xiao WJ He JW Zhang H Yu JB Hu WW. Association of ALOX15 Gene Polymorphisms With Obesity-Related Phenotypes in Chinese Nuclear Families With Male Offspring. Acta Pharmacol Sin (2012) 33:201–7. doi: 10.1038/aps.2011.167 PMC4010344 22301860

[26] Xiao WJ He JW Zhang H Hu WW Gu JM Yue H. ALOX12 Polymorphisms Are Associated With Fat Mass But Not Peak Bone Mineral Density in Chinese Nuclear Families. Int J Obes (Lond) (2011) 35:378–86. doi: 10.1038/ijo.2010.157 PMC3061002 20697415

[27] Xiao WJ Ke YH He JW Zhang H Yu JB Hu WW. Polymorphisms in the Human ALOX12 and ALOX15 Genes Are Associated With Peak Bone Mineral Density in Chinese Nuclear Families. Osteoporos Int (2012) 23:1889–97. doi: 10.1007/s00198-011-1835-3 22089472

[28] Yue H He JW Zhang H Wang C Hu WW Gu JM. Contribution of Myostatin Gene Polymorphisms to Normal Variation in Lean Mass, Fat Mass and Peak BMD in Chinese Male Offspring. Acta Pharmacol Sin (2012) 33:660–7. doi: 10.1038/aps.2012.12 PMC4010361 22426697

[29] Yue H He JW Zhang H Hu WW Hu YQ Li M. No Association Between Polymorphisms of Peroxisome [Corrected] Proliferator-Activated Receptor-Gamma Gene and Peak Bone Mineral Density Variation in Chinese Nuclear Families. Osteoporos Int (2010) 21:873–82. doi: 10.1007/s00198-009-1028-5 19644638

[30] Zhao F Gao LH Li SS Wei ZY Fu WZ He JW. Association Between SNPs and Haplotypes in the METTL21C Gene and Peak Bone Mineral Density and Body Composition in Chinese Male Nuclear Families. J Bone Miner Metab (2017) 35:437–47. doi: 10.1007/s00774-016-0774-7 27628047

[31] Stephens M Scheet P. Accounting for Decay of Linkage Disequilibrium in Haplotype Inference and Missing-Data Imputation. Am J Hum Genet (2005) 76:449–62. doi: 10.1086/428594 PMC1196397 15700229

[32] Barrett JC Fry B Maller J Daly MJ. Haploview: Analysis and Visualization of LD and Haplotype Maps. Bioinformatics (2005) 21:263–5. doi: 10.1093/bioinformatics/bth457 15297300

[33] Abecasis GR Cardon LR Cookson WO. A General Test of Association for Quantitative Traits in Nuclear Families. Am J Hum Genet (2000) 66:279–92. doi: 10.1086/302698 PMC1288332 10631157

[34] Deng FY Liu MY Li MX Lei SF Qin YJ Zhou Q. Tests of Linkage and Association of the COL1A2 Gene With Bone Phenotypes' Variation in Chinese Nuclear Families. Bone (2003) 33:614–9. doi: 10.1016/S8756-3282(03)00234-5 14555266

[35] Lau HH Ng MY Cheung WM Paterson AD Sham PC Luk KD. Assessment of Linkage and Association of 13 Genetic Loci With Bone Mineral Density. J Bone Miner Metab (2006) 24:226–34. doi: 10.1007/s00774-005-0676-6 16622736

[36] McIntyre LM Martin ER Simonsen KL Kaplan NL. Circumventing Multiple Testing: A Multilocus Monte Carlo Approach to Testing for Association. Genet Epidemiol (2000) 19:18–29. doi: 10.1002/1098-2272(200007)19:1<18::AID-GEPI2>3.0.CO;2-Y 10861894

[37] Luo J Zhou W Zhou X Li D Weng J Yi Z. Regulation of Bone Formation and Remodeling by G-Protein-Coupled Receptor 48. Development (2009) 136:2747–56. doi: 10.1242/dev.033571 PMC2730404 19605502

[38] Freemantle N Holmes J Hockey A Kumar S. How Strong Is the Association Between Abdominal Obesity and the Incidence of Type 2 Diabetes. Int J Clin Pract (2008) 62:1391–6. doi: 10.1111/j.1742-1241.2008.01805.x PMC2658023 18557792

[39] Clasey JL Bouchard C Teates CD Riblett JE Thorner MO Hartman ML. The Use of Anthropometric and Dual-Energy X-Ray Absorptiometry (DXA) Measures to Estimate Total Abdominal and Abdominal Visceral Fat in Men and Women. Obes Res (1999) 7:256–64. doi: 10.1002/j.1550-8528.1999.tb00404.x 10348496

[40] Després JP Lemieux I Bergeron J Pibarot P Mathieu P Larose E. Abdominal Obesity and the Metabolic Syndrome: Contribution to Global Cardiometabolic Risk. Arterioscler Thromb Vasc Biol (2008) 28:1039–49. doi: 10.1161/ATVBAHA.107.159228 18356555

