# Supplementary material for: LGR4 Gene Polymorphisms Are Associated With Bone and Obesity Phenotypes in Chinese Female Nuclear Families
Source: Front Endocrinol (Lausanne). 2021 Oct 11;12:656077. doi: 10.3389/fendo.2021.656077 (PMC8544421; doi:10.3389/fendo.2021.656077)
Supplement: Supplementary file 1 [file Table_1.pdf]

Supplemental Table 1. QTDT results for the associations between the single nucleotide polymorphisms and phenotypic variations.

Genotype	rs1531557	rs2219783	rs7927234	rs4514364	rs4923445	rs11030016	rs12796247
Test for population stratification							
Femoral neck BMD	0.794	0.633	0.347	0.432	0.157	0.758	0.544
Total hip BMD	0.625	0.721	0.400	0.533	0.253	0.901	0.471
Lumber spine BMD	0.241	0.374	0.680	0.140	0.401	0.257	0.714
TFM	0.564	0.085	0.091	0.731	0.379	0.083	0.089
TLM	0.895	0.682	0.732	0.703	0.191	0.832	0.604
PFM	0.664	0.160	0.187	0.866	0.648	0.082	0.108
PLM	0.648	0.167	0.198	0.723	0.910	0.054	0.056
Trunk fat mass	0.535	0.097	0.089	0.540	0.546	0.037	0.056
BMI	0.856	0.333	0.309	0.400	0.748	0.159	0.233
Tests for total association							

Femoral neck BMD	0.223	0.521	0.492	0.293	0.705	0.110	0.493
Total hip BMD	0.267	0.566	0.554	0.474	0.942	0.057	0.967
Lumber spine BMD	0.841	0.615	0.901	0.972	0.838	0.348	0.365
TFM	0.379	0.427	0.496	0.858	0.225	0.939	0.894
TLM	0.342	0.727	0.601	0.482	0.375	0.291	0.757
PFM	0.413	0.331	0.447	0.982	0.458	0.774	0.869
PLM	0.501	0.351	0.502	0.837	0.423	0.963	0.970
Trunk fat mass	0.308	0.456	0.477	0.939	0.354	0.898	0.7615
BMI	0.321	0.523	0.501	0.715	0.070	0.874	0.584
Test for within-family association							
Femoral neck BMD	0.492	0.886	0.904	0.191	0.489	0.373	0.953
Total hip BMD	0.642	0.855	0.912	0.343	0.465	0.215	0.592
Lumber spine BMD	0.334	0.811	0.854	0.318	0.669	0.882	0.703

TFM	0.302	0.079	0.099	0.712	0.788	0.237	0.181
TLM	0.573	0.595	0.538	0.436	0.792	0.378	0.873
PFM	0.378	0.095	0.146	0.925	0.817	0.149	0.207
PLM	0.427	0.104	0.172	0.698	0.613	0.157	0.179
Trunk fat mass	0.243	0.097	0.097	0.535	1.000	0.170	0.114
BMI	0.412	0.258	0.236	0.402	0.289	0.257	0.215
P1000 permutation of within-family							
Femoral neck BMD	0.472	0.881	0.893	0.147	0.518	0.419	0.952
Total hip BMD	0.623	0.853	0.900	0.302	0.467	0.237	0.625
Lumber spine BMD	0.349	0.839	0.856	0.307	0.688	0.893	0.715
TFM	0.419	0.168	0.200	0.772	0.776	0.297	0.256
TLM	0.586	0.633	0.567	0.478	0.779	0.387	0.873
PFM	0.419	0.085	0.137	0.928	0.786	0.147	0.174

PLM	0.460	0.090	0.181	0.679	0.608	0.158	0.165
Trunk fat mass	0.258	0.120	0.114	0.490	0.996	0.166	0.090
BMI	0.461	0.308	0.268	0.438	0.281	0.287	0.239

Supplemental Table 2. QTDT results for the associations between the haplotype and phenotype variations.

	block 2			block 3		block 4			block 5
	GAC	AGG	AAG	AACGT	AAAGC	CTA	TTG	TCA	TGTCTC
Test for population stratification									
Femoral neck BMD	0.509	0.559	0.269	0.199	0.849	0.488	0.203	0.079	0.293
Total hip BMD	0.958	0.809	0.564	0.431	0.410	0.688	0.247	0.214	0.397
Lumbar spine BMD	0.246	0.356	0.518	0.335	0.530	0.150	0.428	0.128	0.788
TFM	0.114	0.605	0.059	0.702	0.547	0.544	0.538	1.000	1.000
TLM	0.354	0.676	0.634	1.000	0.829	0.533	0.240	0.234	0.463
PFM	0.179	0.824	0.127	0.673	0.486	0.756	0.850	0.792	0.585
PLM	0.210	0.534	0.151	0.617	0.764	0.603	0.862	0.764	0.771
Trunk fat mass	0.171	0.518	0.052	0.907	0.870	0.363	0.723	0.892	0.663
BMI	0.199	0.233	0.216	0.892	0.922	0.221	0.953	0.600	0.794

Tests for total association									
Femoral neck BMD	0.132	0.854	0.947	0.161	0.086	0.135	0.663	0.935	0.186
Total hip BMD	0.109	0.993	0.827	0.211	0.114	0.337	0.842	0.899	0.250
Lumbar spine BMD	0.134	0.879	0.560	0.245	0.155	0.817	0.882	0.614	0.098
TFM	0.725	0.257	0.498	0.839	0.928	0.542	0.186	0.164	0.963
TLM	0.183	0.355	0.806	0.932	0.274	0.411	0.409	0.564	0.276
PFM	0.898	0.561	0.359	0.673	0.411	0.717	0.398	0.259	0.754
PLM	0.707	0.446	0.350	0.779	0.774	0.574	0.359	0.340	0.920
Trunk fat mass	0.448	0.242	0.401	0.617	0.768	0.487	0.297	0.232	0.623
BMI	0.459	0.249	0.514	0.627	0.503	0.514	0.060	0.111	0.832
Test for within-family association									
Femoral neck BMD	0.121	0.793	0.469	0.058	0.234	0.116	0.586	0.212	0.776
Total hip BMD	0.228	0.866	0.812	0.150	0.082	0.326	0.521	0.350	0.763

Lumbar spine BMD	0.059	0.609	0.974	0.133	0.135	0.258	0.668	0.510	0.288
TFM	0.364	0.237	0.070	0.877	0.634	0.390	0.606	0.301	1.000
TLM	0.819	0.336	0.610	0.972	0.332	0.304	0.844	0.693	0.762
PFM	0.285	0.562	0.085	0.967	0.282	0.633	0.621	0.315	0.892
PLM	0.241	0.326	0.094	0.846	0.680	0.444	0.428	0.366	0.903
Trunk fat mass	0.566	0.191	0.052	0.775	0.785	0.266	0.590	0.457	0.503
BMI	0.720	0.097	0.181	0.812	0.667	0.190	0.193	0.430	0.739
P1000 permutation of within-family									
Femoral neck BMD	0.109	0.785	0.486	0.091	0.251	0.086	0.605	0.207	0.765
Total hip BMD	0.237	0.864	0.785	0.179	0.094	0.280	0.523	0.363	0.753
Lumbar spine BMD	0.075	0.616	0.975	0.185	0.140	0.240	0.700	0.526	0.327
TFM	0.367	0.23	0.174	0.890	0.630	0.496	0.623	0.372	0.990
TLM	0.827	0.354	0.651	0.971	0.370	0.329	0.833	0.710	0.829

PFM	0.341	0.531	0.086	0.959	0.215	0.628	0.603	0.315	0.892
PLM	0.270	0.267	0.100	0.842	0.621	0.452	0.426	0.354	0.910
Trunk fat mass	0.558	0.150	0.082	0.881	0.909	0.266	0.601	0.459	0.514
BMI	0.695	0.104	0.244	0.793	0.641	0.241	0.194	0.470	0.751
